# Development of a knowledge translation taxonomy in the field of health prevention: a participative study between researchers, decision-makers and field professionals

**DOI:** 10.1186/s12961-020-00602-z

**Published:** 2020-08-15

**Authors:** Aurélie Affret, Ollivier Prigent, Marion Porcherie, Olivier Aromatario, Linda Cambon

**Affiliations:** 1 Chaire de prévention ISPED/SPF, Université de Bordeaux, Bordeaux, France; 2 Centre Inserm Université de Bordeaux U1219, BPH, Bordeaux, France; 3 Arènes-Rennes 1 UMR CNRS 6051, EHESP, Rennes, France

**Keywords:** Knowledge translation, taxonomy, public health, health prevention, health promotion

## Abstract

**Objectives:**

The current literature lacks a detailed and standardised description of public health knowledge translation (KT) activities designed to be applied at local levels of health systems. As part of an ongoing research project called the *Transfert de connaissances en regions* (TC-REG project), we aim to develop a local KT taxonomy in the field of health prevention by means of a participative study between researchers, decision-makers and field professionals. This KT taxonomy provides a comparative description of existing local health prevention KT strategies.

**Methods:**

Two methods were used to design a participative process conducted in France to develop the taxonomy, combining professional meetings (two seminars) and qualitative interviews. The first step involved organising a seminar in Paris, attended by health prevention professionals from health agencies in four regions of France and regional non-profit organisations for health education and promotion. This led to the drafting of regional KT plans to be implemented in the four regions. In a second step, we conducted interviews to obtain a clear understanding of the KT activities implemented in the regions. Based on data from interviews, a KT taxonomy was drawn up and discussed during a second seminar.

**Results:**

Our work resulted in a KT taxonomy composed of 35 standardised KT activities, grouped into 11 categories of KT activities, e.g. dissemination of evidence, support for use of evidence through processes and structures, KT advocacy, and so on.

**Conclusions:**

The taxonomy appears to be a promising tool for developing and evaluating KT plans for health prevention in local contexts by providing some concrete examples of potential KT activities (advocacy) and a comparison of the same activities and their outcomes (evaluation).

## Introduction

Emerging evidence points to a consensual need for more evidence-informed public health knowledge translation (KT) practices given the opportunities arising from this kind of approach, e.g. improvement in the efficiency, credibility and sustainability of health systems [1]. In France, the *Transfert de connaissances en regions* (Regional knowledge transfer or the TC-REG) project was set up in order to enhance evidence-based practices within a specific field, namely that of local health prevention policies. The TC-REG protocol has already been published [2]. Briefly, TC-REG is a comparative multiple case study of a KT plan in the field of health prevention, based on a realistic approach [3, 4]. The project is designed to introduce activities aimed at improving the conversion of research-based knowledge into health prevention-related decision-making and practices in four regions in France and to assess their impact through a realistic evaluation [2]. While the process of converting research-based knowledge into decision-making and practice has been given several names in the literature [5, 6] (Table 1 details the different terms), we adopt the most commonly used term, namely, KT. The National Public Health Institute of Quebec defines KT as “*the group of activities and interaction mechanisms that foster the dissemination, adoption and appropriation of the most up-to-date knowledge possible for use in professional practice and in healthcare management*” [7].

**Table 1:** Definitions of terms used

Label	Definition	Example
KT activity	The work of a person/a group/an organisation in order to achieve KT	Development of a multi-professional working group to support field professionals to develop promising actions
KT standardised activity	Standardised labelling for KT activities named differently but that aim the same objective through the same process	Creation or reinforcement of a service/unit/support centre for KT development
Category of KT activities	A family of different KT activities that aim the same objective	Support to evidence use through process and structures (institutional reorganisation to the advantage of KT and EIDM)
KT scheme	A list of KT activities that can be implemented in local contexts	Fig. 1 details a KT scheme
KT pragmatic category	A family of different categories of KT activities that aim the same objective	To improve organisations and processes in order to facilitate the integration of knowledge
KT strategy/intervention/initiative	Everything that can be done in order to improve KT	It can be a KT activity, a KT standardised activity, a category of KT activities, a KT scheme or a KT pragmatic category
KT taxonomy or KT classification scheme	A structured way of classifying KT activities	Table 4 details a KT taxonomy
KT process	The path of how a KT activity can produce an effect	Knowledge creation [5]
KT framework	A map that structures KT processes	Knowledge to Action framework [5]

*EIDM* evidence-informed decision-making, *KT* knowledge translation

Many scholars have highlighted the challenge of converting research-based knowledge into evidence-informed practices and decision-making in the public health field [8, 9]. First, barriers linked to people, organisations, contexts and properties of the evidence persist [10–12], preventing the optimal production and use of evidence [13–15]. Second, the literature on KT provides many frameworks and taxonomies [16–19], but these are not always adapted to the needs and practices of health prevention professionals, including decision-makers. Indeed, most of them deal with generalist frameworks, such as those described by Milat et al. [20] (e.g. KTA and PARISH), omitting to mention some KT specific activities or whether any taxonomies exist for them. These generalist frameworks are mostly (1) healthcare focused (e.g. nursing, obesity treatment) [21, 22], (2) patient focused [23], (3) strategy focused [22] or (4) objective or mechanism focused [22, 24] (e.g. healthcare professionals’ practices are validated and the criteria are therefore patient oriented only, whereas health prevention requires a more comprehensive approach, including practices that help identify and determine solutions to address potential barriers to evidence-based practice). We can nonetheless mention two major studies that highlight some evidence-based activities [12, 25] in KT. These studies are very helpful to clarify concepts and define methods but, after review, present strategies that are not always adapted to local contexts nor to the field of health prevention outside the care setting. Indeed, the field presents several characteristics that are different from care settings, that is, there is not always clear evidence of practices, the actions and policies are often performed by non-profit organisations, and professionals or volunteers working in these organisations are not always trained in evidence-based practices and they work with few resources. It was also difficult to use both studies in TC-REG without specific appropriation by stakeholders in the different settings or the identification of specific activities, recognised as effective and feasible on the ground. Moreover, the activities need to be specifically described to ensure that they are indeed the activities described in the taxonomy. In effect, we observed that this field of health prevention often uses different terms to describe the same activities (with potentially the same effect) or else the same term is used to talk about different activities (with potentially different effects). Moreover, some activities, such as training, methodological support and knowledge brokering, can be described with the same words in different frameworks, when in fact they are different activities that trigger different mechanisms. For instance, a short training course may only raise awareness of the interest of evidence-based decision-making, while long training courses can include skills for analysing and transferring evidence-based action. The former can enhance the incentive to use evidence, while the latter can provide the skills required to apply it. In this paper, we describe an empirical process we adopted to develop a KT taxonomy that helps to clarify variations in potential KT activities in local health prevention policies. We used a participative approach between researchers, decision-makers and field professionals involved in the KT research project TC-REG. TC-REG, which is still ongoing, began in 2017 and is designed to assess a KT plan to improve policy-making and practices for implementing health prevention in French regions [2]. We argue that this kind of taxonomy can provide operational guidance to local health authorities in order to implement, evaluate and compare KT activities in the field of local health prevention and thus strengthens the evidence in this field.

## Methods

### The TC-REG study

The TC-REG study aims to test and characterise the facilitators that enable public health stakeholders to address the challenges of KT, incorporating academic health prevention knowledge into policy and practice. To this end, we developed a participatory study that involved participants as co-researchers. This means that all the different stakeholders are involved at all stages, including in their development. The participants were those whose work informed the research and who had an influence over the research process. Thus, decision-makers from regional health agencies (*Agence Régionale de Santé*; ARS) and field professionals from non-profit organisations (*Instance Régionale d’Education et de Promotion de la Santé* (Regional Authority of Education and Health Promotion); IREPS) were involved in the study. ARS are responsible for policy-making and health prevention policies. IREPS, which are non-profit organisations, develop health promotion and health prevention programmes and provide methodological support to field professionals in the implementation of health prevention schemes in different settings (e.g. workplaces, schools, care settings, recreation and community centres, rural and urban areas). ARS and IREPS work together to implement health prevention and health policies in local contexts. The TC-REG plan has been rolled out in four French regions to date.

### Study design

The aim was to develop a taxonomy for a KT plan in the field of local health prevention. This study is embedded in the TC-REG research project and unfolds in two stages. First, we organised a seminar with ARS and IREPS professionals involved in the TC-REG project. This was designed to identify the most feasible and best KT activities to implement in the four regions involved in the project (Step 1). The activities selected were embedded in four KT plans adapted to local contexts, one per region, over 12 months. Interviews were then conducted in the regions to assess the nature and purpose of the activities currently in place as precisely as possible in order to enhance evidence-based decision-making/action. Informed by these data, a KT taxonomy was subsequently developed (Step 2).

#### Step 1: Seminar with ARS and IREPS professionals to develop contextualised KT plans

##### Preparing the seminar

To prepare the seminar, in addition to the literature presented in the discussion, we analysed a major piece of evidence [25] published in 2016. We chose it because it is relatively recent and combines a systematic review of the evidence-informed decision-making (EIDM) literature and an extensive review of the research reported in the broader social science literature.

The aim of the study was to identify effective strategies to overcome barriers to EIDM that would fit in with our own aims. The first part was designed to identify the best ways to increase EIDM, while the second part identified insights from social science knowledge to support its use. the authors grouped the interventions reviewed in accordance with six processes by which EIDM might be achieved [25], namely (1) awareness, defined as building awareness of and positive attitude toward EIDM; (2) agreement, defined as building mutual understanding and agreement on policy-relevant issues and the kind of evidence needed to resolve them; (3) communication and access, defined as providing communication of and access to evidence; (4) interaction, defined as interaction between decision-makers and researchers; (5) skills, defined as supporting decision-makers to develop skills in accessing and making sense of evidence; and (6) structure and process, defined as influencing decision-making structures and processes.

We performed an analysis of these studies in order to identify the conditions of KT intervention effectiveness and the expected outcomes of each intervention. This analysis, based on a common framework, was processed independently by three researchers. The findings were compared and discussed by the three researchers in two meetings until consensus was reached. Thus, for each intervention, its definition and description, the conditions of its effectiveness (either by itself or in combination with other interventions), and its influence on the use of evidence were described. The plan was to complement the findings with other sources dedicated to KT interventions in the public health sector at regional or local policy-making and planning level, as presented in the introduction.

Analysis of Langer’ document [25] provided an overview of the effective interventions and categories involved in EIDM. These included a list of effective KT strategies and a list of mechanisms expected to be triggered by them. We grouped the six processes described in Langer’s work [25] into three pragmatic categories of KT activities, as follows: (1) providing access and adaptation of knowledge (pragmatic KT category 1), (2) developing skills and capabilities to analyse, adapt and translate evidence into practice (pragmatic KT category 2), and (3) restructuring working environments to facilitate EIDM (pragmatic KT category 3). For each of these categories, we highlighted the main activities likely to be effective as reported in the work of Langer et al. [25]. In total, nine main activities likely to be effective according to the three categories were presented in the first seminar. The classification is presented in Table 2; the most effective types of activities are also highlighted along with a brief description. Three main activities were considered relevant to providing access to and adaptation of knowledge (pragmatic KT category 1), namely internal and external advocacy, adaptation of communication techniques, and adaptation of dissemination techniques. The most effective types of activity were (1) use of anti-marketing, (2) public segmentation in order to provide appropriate communication, (3) formulation of messages concerning the profit/loss ratio, (4) explanation of uncertainty, (5) use of accounts, records, metaphors and analogies, (6) online media and social networks, (7) labelling strategies, (8) reminders, memory aids, notebooks, and (9) needs-centred communication (Table 2). Two main activities were found to be useful in developing professionals’ skills to analyse, adopt and transfer knowledge into different contexts (pragmatic KT category 2), namely field professional/researcher interactions and training courses. The types of activities that appeared to be most effective were (1) reading clubs, (2) mentoring/guidance to develop evidence-based interventions, (3) training in line with andragogy principles, (4) e-learning, (5) supervision-related training courses, and (6) tailored training content (Table 2). Four activities were found to be useful to improve the organisation and processes in order to facilitate knowledge implementation (pragmatic KT category 3), namely creation/modification of social and professional norms to promote the use of evidence (to make the EIDM the decision-making principle), facilitation, collaboration, and participatory management (Table 2). The types of activities that appeared to be most effective were (1) social marketing techniques, (2) social incentives (norms of use), and (3) facilitation tools (Table 2).

**Table 2:** The effective KT activities described in the work of Langer et al. [25] grouped into three KT pragmatic categories

***KT activities***	***Types of KT activities***	***Description***
**KT pragmatic category 1: To provide access to knowledge and adaptation**		
Internal and external advocacy	• Use of anti-marketing^a^: to highlight the possible negative effects associated with non-access/non-use of evidence • Use of accounts, records, examples • Use of simulations: to model, via a software or a real simulation, the effects of evidence-informed policies vs. non-evidence-informed policies	• Increasing the visibility and credibility of EIDM using several emotions-based strategies: humour, surprise, anxiety, etc. • Decreasing cognitive barriers related to behaviour change and evidence use in decision-making
Adaptation of communication techniques	Public segmentation in order to provide an appropriate communication^a^	Targeting and adapting communication according to homogeneous groups (characteristics, needs, preferences, schedules, familiarity with research), i.e. differentiated, targeted and personalised messages according to targets using user-friendly designs
	Formulation of messages considering the profits to loss ratio^a^	Displaying evidence in order to highlight the profits and loss from using it
	Formulation of norms/identities	Matching EIDM communication with decision-makers norms/identities; evidence is presented from decision-makers point of view
	Explication about uncertainty^a^	Using techniques to explain uncertainty in order to reduce the ambivalence in research results
	Use of accounts, records, metaphors, analogies^a^	Using accounts, records, metaphors, analogies to render evidence accessible (it produces engagement and identification)
Adaptation of diffusion techniques	Online media and social networks^a^	Increasing communication through several strategies: database, contextualised messages, etc.; it is a question of developing personalised app’s (algorithms related to decision-makers’ needs) in order to render EIDM well-known (through its practical aspect)
	Label strategies^a^	Developing a label visibility (logo, slogan, promotional material) related to evidence use; it produces an emotional link with EIDM
	Reminders, aide-mémoire, notebooks^a^	Using recall and memorisation techniques in the case of a communication/intervention on research results (regularity)
	Synchronisation	Using communication when decision-makers are the most receptive (early in the decision-making process, during media coverage, etc.)
	Needs-centred communication^a^	Using understandable communications that agree wholeheartedly with decision-makers and that enable them to positively imagine research results.
**KT pragmatic category 2: To develop professionals’ skills to analyse, adopt and transfer knowledge into their contexts**		
Stakeholders/researchers interactions	Areas to discuss on evidence (journal club), including reading clubs^a^	Implementing formative interventions to share and discuss scientific results (applicability, interest, etc.), to develop a professional consensus on evidence and adapt it to practice; it is also question of adapting evidence to real life: to combine evidence communication with applicability and feasibility communication, for example
	Mentoring/guidance to develop evidence-based interventions^a^	Improving existing mentoring actions considering the changes in professional and behaviour norms; interactions are used as a tool to develop these norms
	Inter-professional education (shared formation)	Using multidisciplinary and interactive learning (decision-makers and other evidence-users, decision-makers and researchers, etc.) through a formative process that clarifies representations from each other; it increases consensus and tolerance to points of view; it enables the modification of evidence perceptions, for example, it is possible to use communities of practice, e.g. mutual learning and experiences sharing interventions between physicians; it could be used to develop a consensus on practices and professional standards, e.g. debates on the interest of evidence in order to increase the opportunity to use it; it is a question of developing shared professional norms through a shared formation process
Formations	Formations to evidence critical analysis and use according to andragogy principles^a^	Increasing EIDM capacities using validated theories for adult formation (andragogy) in order to result in a more rewarding and effective experience in formation (acting on the motivation and capacity); it is also a question of influencing attitudes towards evidence, through EIDM formation, in order to render attitudes positive
	E-learning^a^	Increasing data availability and convenience for data use
	Formation to supervision^a^/organisation of evidence use in the organisations	Training experienced decision-makers with the aim to increase their skills to supervise evidence use in their teams; supervision supplies encouragement to apply their EIDM expertise (motivation); it gives the opportunity to use the skills learned
	Personalisation of formation contents^a^ by the inclusion of experiential and contextual data (improvement of formation process)	Improving EIDM formations thanks to real life and contextual data (to increase the opportunity to use evidence) and frequently repeating the formations focusing on the most relevant and individualised content/skills (to increase the capacity and motivation to use evidence) Mentoring/guidance can be used for interventions to be based on evidence
**KT pragmatic category 3: To improve organisations and processes in order to facilitate the integration of knowledge**		
Creation/modification of social and professional norms to lead to evidence use (to render EIDM the decision-making principle)	Nudges	Supporting decision-makers to use evidence by re-thinking choices (formulation of options to choose) in order to increase evidence use
	Social marketing techniques^a^	Using social marketing techniques, including definition of a behaviour on which it is possible to act, public segmentation (public homogeneity), marketing techniques management, discussion on benefits and costs related to behaviour change, barriers identification
	Interventions in order to support an identity rapprochement	Highlighting the connexion of people with social identity or norm, in order to increase the collective norm; it helps to start and increase the growing norms of evidence use, to institute evidence use as an identity norm (in a structure for example); habits, practices, vision and reputation in accordance with evidence use have to be thought and used
	Social incentives (norms of use)^a^ – evaluation, recognition, valorisation, etc.	Using non-financial incentives, with social value, in order to give to evidence use the status of behaviour norm to increasingly conform people to use it
Facilitation	Facilitation tools^a^	Using tools that support evidence use, for example, audits, feedbacks, financial or professional incentives, tools that assist in decision-making
	System of knowledge management	Improving and making knowledge exchanges formal inside the organisation
	Knowledge brokering	Knowledge brokers directly work with stakeholders in order to highlight topics for which evidence data would be useful
Collaboration	Learning organisations	Developing environments in which decisions could be questioned, challenged and informed via evidence data
	Networking	Informally organising a group of decision-makers and/or researchers interested in EIDM as if they were mere users of information technologies
	Projects of collaborative research	Helping decision-makers to identify research issues and be aware of the importance of evidence use in order to help them to find solutions to these issues; evidence users need to be included in evidence production in order to increase their understanding of what is the research and the perception of its value; a collaboration that renders evidence ‘practical’ needs to support this
Participatory management	Management techniques using social psychology and communication in order to obtain a consensus on evidence use	Concerted planning, consultation, etc.; some leadership techniques (e.g. the egalitarian technique or the transformational technique) and some management techniques (e.g. adaptative) give adequate organisational conditions for EIDM use
	Feedback	Challenging the status quo and provoking a debate on evidence use; the two techniques the most used are the redteaming (to study a topic from another point of view) and the dogfooding (to use its own services in order to detect their qualities and defaults); it is question of giving an opportunity for decision-makers and beneficiaries of their decisions, to express their opinion and contest evidence use and its adaptation: meetings feedbacks, experience feedbacks, restructuration of organisations, feedback forms, etc.

*EIDM* evidence-informed decision making, *KT* knowledge translation

^a^Types of KT activities that appeared to be the most effective

##### Conducting participative seminars

We then organised the seminar in order to develop a contextualised KT plan for each region based on the best KT activities. The KT plans included KT activities to be implemented and the expected outcomes.

We organised a participative 2-day seminar with the researchers and professionals involved in the TC-REG project with all the ARS and IREPS, each represented by one or two members. In addition, two researchers who were specialists in KT and realistic evaluation were appointed as consultants to support the process. In total, 17 professionals took part.

The seminar was split into four stages based on the participants’ involvement in round tables and working sub-groups. First, the basics of KT processes and tools were presented in order to raise the awareness of participants in the field. This stage provided an opportunity for the regions to describe and talk about the KT initiatives that already existed during a specific round table. In a second stage, certain ‘knowledge documents’ (*Stratégies d’Intervention en Prevention*, i.e. intervention strategies in health prevention) were presented. The documents were specifically created for the TC-REG project and provide evidence of effective health prevention strategies in five priority areas in France, namely nutrition, alcohol, tobacco smoking, emotional and sexual health, and psychosocial skills [26–30]. The documents are based on systematic reviews and international guidelines. In the third stage, the participants were split into two groups, each from two regions. Based on the two supporting documents drawn up by the most appropriate KT strategies and actions from Langer et al.’s [25] work to implement in French and local contexts, informed by two criteria, the three strategies defined in the preparatory process were combined with the best evidence-based activities. Each group was asked to choose activities from the three categories and to explain the form they could take in different contexts. In the fourth stage, the participants were split into four regional groups and asked to define the activities they would like to and could implement in their own region in accordance with the strategies listed, their needs and resources, and to formulate hypotheses about the effects/outcomes expected in terms of evidence-informed practices. This led us to define four specific KT plans, one per region, describing the activities and expected outcomes adapted to the different health prevention professionals such as IREPS professionals, ARS professionals, stakeholders/field professionals and professionals from advisory organisms involved in implementing regional health policies.

**Fig. 1:**
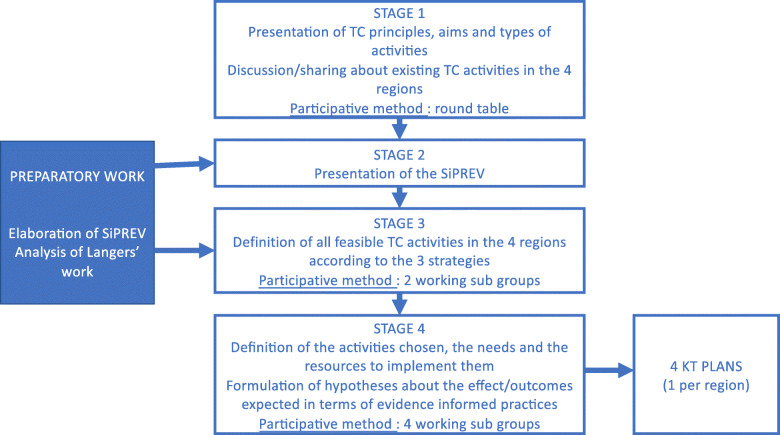
Seminar process

Step 1 was managed by the research team and both guests who were given the KT plans implemented in the four regions by IREPS and ARS during the 12-month health prevention policies and actions period. Figure 1 presents the different stages of step 1.

#### Step 2: interviews and a second seminar

Some discrepancies arose between the planned process and its realisation in real conditions. Indeed, when the four KT plans were designed during the workshop, the feasibility and sustainability conditions still needed to be substantiated. After the KT plans had been implemented in the regions, it was clear that changes could potentially occur according to the resources and existing local initiatives. Once aware of the potential adjustments in order to know exactly which KT activities had in fact been implemented, the research team conducted a qualitative study 3 months after implementation of the KT plans. This was designed to collect data on the KT activities actually adopted in the regions, including their exact description in order to compare and distinguish them in the TC-REG evaluation.

Ten interviews (two or three per region) and one focus group per region were carried out. The semi-structured interviews were conducted by phone by one researcher, each lasting 50–90 min as, in addition to asking professionals about KT activities adopted in the regions, other aspects of the TC-REG project were also investigated. The focus groups were conducted by the same researcher with the participation of 4–6 professionals from each region. The aim was to obtain a consensus on the data collected during the interviews. The professionals interviewed included project managers and TC-REG referees from both institutions (ARS and IREPS) and all four regions. All of the interviews were digitally recorded and transcribed. The data was analysed by thematic analyses using N’Vivo® software. The analyses yielded a list of KT activities implemented in the four regions. Certain activities were given different names in the different regions but aimed for the same goal with the same implementation process. We grouped them with standardised labels (standardised KT activities) and the standardised activities were then put into categories of KT activities (taxonomy V0).

Finally, to adjust and hone the taxonomy V0, it was first discussed during a second 1-day seminar attended by the researchers (here, the authors), decision-makers and field professionals involved in the TC-REG project. Each activity was discussed to ensure it was (1) clearly distinguishable, (2) really implemented and (3) specific (one activity = one purpose). The round table discussion addressed each activity individually. Minor adjustments (essentially semantic) were made, leading us to define the taxonomy V1. Step 2 provided a first consensually agreed KT taxonomy (taxonomy V1).

## Results

### Step 1: the contextualised KT plans

**Fig. 2:**
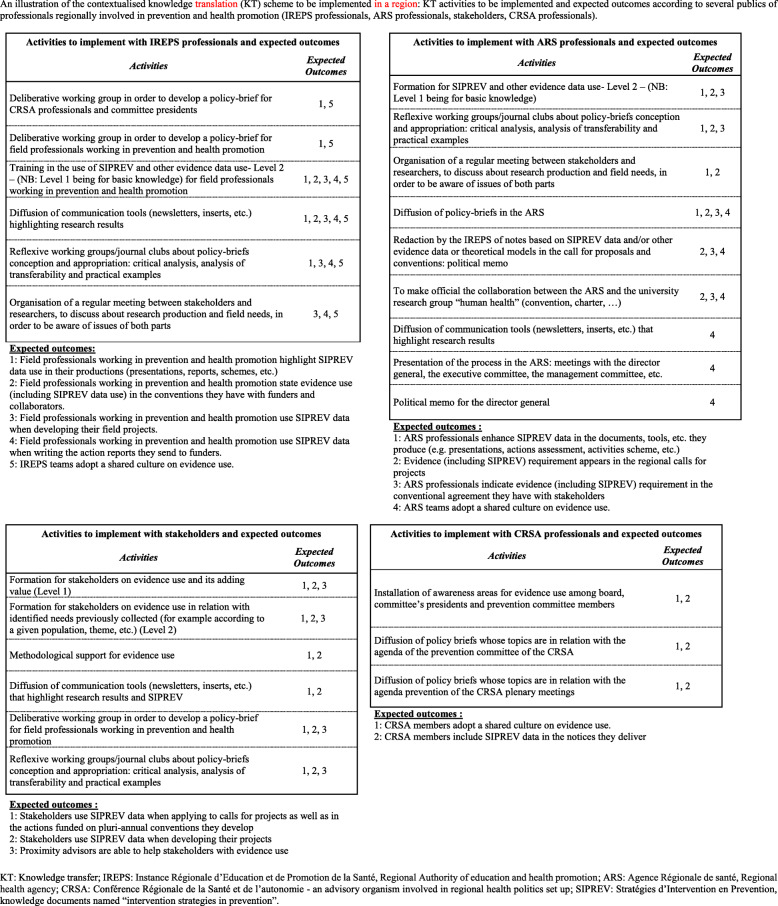
Illustration of the knowledge translation plan for one region

Based on the data set out in Table 2 and according to the regional contexts, four KT plans were developed by the ARS and IREPS professionals involved in the TC-REG project, i.e. one contextualised KT plan per region. The KT plans attempted to combine knowledge access and adaptation (pragmatic KT category 1), with some activities designed to develop professionals’ skills in analysing, adopting and transferring knowledge to their different contexts (pragmatic KT category), while improving the organisations and processes in order to facilitate knowledge integration (pragmatic KT category 3). Figure 2 provides an illustration of the KT plan for one region. In this region, for instance, the KT plan targeted four professional publics: professionals from IREPS, professionals from ARS, stakeholders/field professionals, and professionals from the CRSA (*Conférence Régionale de la Santé et de l’autonomie* – an advisory organism involved in a regional health policies set-up). It included six KT activities implemented with IREPS professionals who targeted five expected outcomes, with nine KT activities implemented with ARS professionals targeting four expected outcomes, six KT activities implemented with stakeholders/field professionals targeting three expected outcomes, and three KT activities implemented with CRSA professionals targeting two expected outcomes (Fig. 2).

### Step 2: a consensually agreed KT taxonomy

The qualitative analysis of the interviews and focus group led to the description and reporting of about 10 to 30 KT activities per region. A V0 taxonomy was then established by the research team. Based on this report and during the second 1-day seminar, the taxonomy was presented and discussed with the participants. A slightly adjusted KT taxonomy V1 was consensually adopted by the researchers, stakeholders and decision-makers involved in the TC-REG project. There was a major change to one activity, highlighted during the seminar but performed in none of the four regions, namely, the ‘methodological support for the standardised KT roll-out’ activity (standardised activity 9.5., Table 3). This was added as it is scheduled to be set up by stakeholders after the study. Taxonomy V1 describes 35 standardised KT activities (one standardised activity = one purpose), grouped into 11 categories of KT activities, namely dissemination of evidence; adaptation of evidence; project identification, image and so on, presenting KT processes so as to be visible to the institutions; evidence-dedicated communication; communication on evidence in communications not dedicated to evidence; training in use of evidence; appropriation of evidence; support for evidence use through processes and structures; methodological support for evidence use; co-construction of KT tools; and KT advocacy. The work is presented in Table 3. Table 4 presents the final general taxonomy, completed by a brief description of each of the KT standardised activities.

**Table 3:** The KT taxonomy developed in a consensual work between decision-makers, field professionals and researchers involved in the TC-REG project based on KT activities implemented in region

Categories of KT activities	Standardised KT activities		KT activities in region 1		KT activities in region 2		KT activities in region 3		KT activities in region 4	
	N°	Description	Description	a	Description	a	Description	a	Description	a
**1 – Diffusion of evidence data** *Diffusion of documents that include evidence data (SIPREV or other evidence data)*	**1.1**	**Paper diffusion of evidence**	Paper diffusion of evidence by CRES documentalists on request of partners	1	Paper diffusion of summarised evidence by IREPS, during several events	1	Paper diffusion of evidence by IREPS, to participants of activities carried out within the framework of the KT support plan (TC-REG) + Distribution of evidence via the IREPS documentation/bibliographic centre	2	Paper diffusion of evidence by IREPS to participants of the reflective workshops conducted within the framework of the KT support plan (TC-REG)	1
	**1.2**	**Diffusion of evidence by email**	Email diffusion of evidence by CRES documentalists on request of partners	1	Email diffusion of evidence to subscribers to IREPS resources according to their themes of interest	1	Email diffusion of evidence to field professionals	1	Email diffusion to field professionals on specific request	1
	**1.3**	**Inclusion of evidence in bibliographic tools**	Referencing of SIPREV and resources used in the framework of the KT support plan (TC-REG) in a bibliographic database produced by the CRES Integration of evidence in all bibliographic selections performed by CRES documentalists	2	More systematical integration of evidence into documentary products (bibliographic selections, syntheses)	1				
	**1.4**	**Diffusion of evidence via websites**	Diffusion of evidence on a secure social network shared by professionals involved in the “Mois Sans Tabac” (French Tobacco-Free Month)	1	Diffusion of SIPREV on a regional documentary portal of documentary and pedagogical resources in education and health promotion	1	Diffusion of evidence via the IREPS website	1	Diffusion of evidence via the IREPS website	1
**2 - Adaptation of evidence** *Transformation of evidence or documents that include evidence in order to render them more intelligible and more specific to some publics/elaboration of new documents or utilisation of existing documents*	**2.1**	**Inclusion of evidence (from SIPREV or not) in usual communication tools**	Inclusion of evidence in newsletters	1					Inclusion of evidence into communication tools (newsletters, inserts, etc.)	1
	**2.2**	**Adaptation and diffusion of evidence elements (SIPREV or others) through video capsules**					Adaptation and diffusion of evidence elements through video capsules	1		
	**2.3**	**Creation of bibliographic selections (evidence-based actions)**	Creation of bibliographic selections (inspired by the SIPREV) in response to all calls for projects or new projects in general	1						
	**2.4**	**Adaptation and diffusion of elements from evidence (SIPREV or others) into policy briefs/explicit and oriented notes/knowledge documents**	Production of a KT document (to promote soft and active mobility in urban areas) for local authorities Response to specific requests from the ARS (syntheses of evidence and identification of evidence-based actions in various sources, including SIPREV)	2	Elaboration of online documentary syntheses (“Vulnerabilities and health”, life skills)	1	Elaboration of guidance notes	1	Elaboration of an administrative note for the director general Creation of policy notes adapted to school environment	2
**3 – Identification of a project, image, etc., gathering KT processes to be visible to institutions** *To identify/use a project/a shared image gathering KT processes, visible image/project for the institution(s)*	**3.1**	**Institutional communication about a KT programme/plan**	Communication on the KT scheme in region, during the seminar to launch this scheme Communication on the KT scheme in region and the associated research in the “priorité santé” journal (health priority journal) Presentation of the KT scheme at the CRES Board of Directors	3	Presentation of the KT scheme in region and the associated research in the CRSA’s specialised prevention committee	1	Presentation of the KT plan at various occasions (meeting to launch the plan, meetings with the director general, meeting with the directive board on the progress of the work and EIDM, meetings with departmental delegations and professional divisions)	1	Presentation of the KT support plan to ARS members during the training sessions planned within the support plan	1
	**3.2**	**Use of the KT programme to develop specific partnerships (research, other associations)**	Partnership with the UNESCO Chair that works with research teams	1						
	**3.3**	**Identification of a graphic charter for KT activities**					Use of the graphic charter of the video capsules in the communication about the KT support plan (in PowerPoint presentations, emails, etc.)	1		
	**3.4**	**Evaluation of its KT strategy**					Review of experiences during the feedback seminar Questionnaire to all participants to evaluate the activities of the KT scheme	2		
**4 – Communication dedicated to evidence** *Planning communication moments specifically dedicated to evidence*	**4.1**	**Symposium/meeting including specific communication about evidence/SIPREV**	Seminar to launch the KT support plan (2 days) Presentation of the SIPREV on nutrition to local and regional authorities Communication and reflection about the tools developed within the framework of the KT support plan	3			Seminar to launch the KT support plan Seminar to present the SIPREV and other materials	2	Reflexive workshops conducted within the framework of the KT support plan Intra-IREPS meeting to present the TC-REG project to the whole team	2
**5 - Communication on evidence in communications not dedicated to evidence** *Planning/realisation of communications on evidence during communication moments not dedicated to evidence*	**5.1**	**Communication/mention of evidence/SIPREV within meeting not dedicated to evidence/SIPREV**			Integration of evidence into a communication on life skills, during a regional day organised for secondary schools by the Local Education Authority	1	Communication on the KT support plan of Normandie during a research symposium on interventional research	1	Presentation of the KT support plan on several occasions (public health meetings in the French Guiana Antilles, presentation to the presidents of the CRSA committee and to the CRSA Prevention Committee)	1
	**5.2**	**Inclusion of the interest of evidence (added value) into existing thematic or generalist training courses**	Request from CRES network trainers to add evidence into existing thematic or generalist training courses	1					Introduction to the interest/adding value of evidence in several training courses (life skills formation, healthcare service formation, ‘addictions’ formation)	1
**6 – Training on evidence use** *Organisation of training sessions on evidence utility, adding value and use*	**6.1**	**Awareness on evidence use (meeting, seminar, etc.)**					Awareness on evidence issues	1	Awareness on evidence issues for field professionals	1
	**6.2**	**Training on evidence/SIPREV analysis and use**					Training on evidence analysis and use	1	Presentation of the KT support plan to ARS members within the ARS training framework	1
**7 – Appropriation of evidence** *Exchange and working sessions that foster the identification, sharing and analysis of evidence (they could lead to the production of tools but not necessarily)*	**7.1**	**Analysis and exchange workshops on evidence/SIPREV**	Working groups (4/6 days) “Thé Santé” (a tea moment to talk about evidence and research results) Inter-professional day of exchange and work on the extension tool for local authorities Day of mobilisation of local authorities	4	Progress points/feedbacks with local partners in the “Précapss” interventional research (development of physical activity for people in sedentary/precarious situations)	1	Reflective workshops (with evidence use planning) Regular exchanges of practices on evidence/sharing on literature with key field professionals or researcher (as part of the KT support plan)	2	Regular sessions of exchange between field professionals and researchers Reflective workshops (with CRSA, field professionals)	2
**8 – Support to evidence use through processes and structures** *Institutional reorganisation to the advantage of KT and EIDM*	**8.1**	**Service/unit/support centre for KT development**	Creation of a support unit within the ARS: to identify innovative projects as well as field professionals/researchers to support those projects using evidence Development of a multi-professional working group to support field professionals to develop promising actions	2			Support for KT development and evidence integration set up by the IREPS internal quality department	1		
	**8.2**	**Service/ unit/ pole for evaluating promising practices**	Creation of a support unit within the ARS: to identify innovative projects and field professionals/researchers to support those projects using evidence Development of a multi-professional working group to support field professionals to develop promising actions	2						
	**8.3**	**Amendment or reinforcement or orientation of the activity of an existing KT plan**	Creation of a popularisation activity on evidence within the “Dispositif Régional de Compétences” (Competence Regional Plan), designed to be renewed from year to year	1			Interventional research and KT areas within the KT support plan	1	Creation of a KT committee in the DAPPS (“Dispositif d’Appui à la Prévention et Promotion de la Santé”, support plan for prevention and health promotion)	1
	**8.4**	**Internal coordination meetings (how to use evidence?)**	Internal CRES meeting to study calls for projects and integrate evidence into them	1	Exchanging practices time during the IREPS’ internal methodological meetings in which evidence is addressed (usefulness, needs in terms of bibliography, etc.)	1	Meetings between thematic and territorial referees	1	Internal meetings	1
	**8.5**	**Reminder of the importance (interest, added value) of using evidence/SIPREV during team and/or project meetings**	Internal meetings organised by the research and innovation department in order to relay the importance of using evidence when writing the conventions with field professionals	1			Reminder of the importance of using evidence during IREPS internal team meetings	1		
	**8.6**	**Reminder of the importance (interest, added value) of using evidence/SIPREV in work or financial documents**	Reminder of the value of using evidence when responding to calls for proposals	1			Integration of the notion of evidence in the calls for proposals of the prevention and health promotion department of the ARS	1	Guidance note on evidence within some calls for proposals	1
**9 – Methodological support to evidence use** *Using specific tools or support field professionals that help to evidence use, analysis and transfer*	**9.1**	**Occasional methodological support including evidence (less than 2 h)**	Methodological support that includes evidence, provided by CRES	1						
	**9.2**	**Short methodological support including evidence (2 to 6 h)**	Methodological support that includes evidence, provided by CRES	1						
	**9.3**	**Long methodological support including evidence (more than 6 h)**	Methodological support that includes evidence, provided by CRES	1						
	**9.4**	**Existence of a proactive referent for KT roll-out: to encourage, mobilise, remind and support KT development**					Existence of a proactive referent for KT roll-out: to encourage, mobilise, remind and support KT development	1		
	**9.5**	**Methodological support for KT roll-out**								
	**9.6**	**Creation and diffusion of methodological tools based on SIPREV (grids, referentials) to support SIPREV use, in an autonomous way**							Elaboration of METISSE forms (“MEs anTI Sèches sur les données issues des Sciences et de l’Expérience”, my help for data from Science and Experience) Reading guide for the SIPREV on life skills	2
	**9.7**	**Development of a methodological guide to help KT implementation**					Development by the IREPS of a KT support guide	1		
	**9.8**	**Development of methodological guides to assist in the use of tools developed using evidence (from SIPREV or not)**	Production of kits to support the diffusion of slides and posters (glossaries, notices, etc.) Development of tools (grids and semi-directive interview questionnaires) to identify promising actions	2						
**10 - Co-construction of KT tools** *Approaches to* *develop new shared knowledge*	**10.1**	**Multidisciplinary and multi-professional co-construction of KT tools and processes**	Working groups to produce ad hoc tools	1	Impulse of interventional research procedures by the IREPS	1				
**11 – KT advocacy** *Implementation of advocacy strategies to support EIDM*	**11.1**	**Advocacy to decision-makers**	Elaboration and diffusion of a popularisation tool on physical activity promotion in urban areas for local authorities	1			Advocacy to decision-makers (with the director general and during the co-direction meetings when talking about the progress of the TC-REG project and the EIDM)	1	Presentation of the KT support plan to the director general of the ARS and the CRSA (President and Prevention Committee) Awareness moments on the use of evidence for the Board, committee presidents and members of the Prevention Committee	2
	**11.2**	**Advocacy to partners**			Advocacy to partners within the framework of the CRSA’s prevention specialised committee (presentation of the ‘life skills’ approach highlighting that it is based on evidence, SIPREV diffusion, presentation of the research project associated to the KT support plan, presentation of easy methods to access to evidence syntheses)	1	Discussion on evidence during several institutional events	1	Presentation of the TC-REG project to the director general of the ARS and the CRSA (President and Prevention Committee)	1

*ARS* Agence Régionale de santé (Regional health agency), *CRES* Comité regional d’éducation pour la santé (Regional authority of health education: IREPS equivalent in PACA), *CRSA* Conférence Régionale de la Santé et de l’autonomie (an advisory organism involved in regional health politics set-up), *EIDM* Evidence-informed decision-making, *IREPS* Instance Régionale d’Education et de Promotion de la Santé, Regional Authority of education and health promotion, *KT* knowledge translation, *SIPREV* Stratégies d’Intervention en Prevention (knowledge documents named ‘intervention strategies in prevention’)

^a^Number of activities implemented in region

**Table 4:** The final generalist KT taxonomy completed by a brief description of each KT activities

Categories of KT activities	Standardised KT activities		Definition
	N°	Description	
**1 – Diffusion of evidence** *Diffusion of documents that include evidence*	**1.1**	**Paper diffusion of evidence**	Documents that include evidence (e.g. SIPREV, other evidence, summarised evidence, etc.) are distributed in paper format
	**1.2**	**Diffusion of evidence by email**	Documents that include evidence (e.g. SIPREV, other evidence, summarised evidence, etc.) are sent via e-mail
	**1.3**	**Inclusion of evidence in bibliographic tools**	Evidence is included in bibliographic tools (e.g. bibliographic selections, syntheses, etc.)
	**1.4**	**Diffusion of evidence via websites**	Evidence is included in some websites (e.g. institutional websites, partners’ websites, field professionals’ structures websites, etc.)
**2 - Adaptation of evidence** *Transformation of evidence or documents that include evidence in order to render them more intelligible and more specific to some publics/elaboration of new documents or utilisation of existing documents*	**2.1**	**Inclusion of evidence in usual communication tools**	Inclusion of evidence in usual communication tools (newsletters, inserts, etc.)
	**2.2**	**Adaptation and diffusion of evidence elements through video capsules**	Adaptation and diffusion of evidence elements through video capsules
	**2.3**	**Creation of bibliographic selection (evidence-based actions)**	Creation of bibliographic selections when responding to calls for projects, developing new projects, etc. in order to set up evidence-based actions
	**2.4**	**Adaptation and diffusion of elements from evidence data into policy briefs/explicit and oriented notes/knowledge documents**	Adaptation and diffusion of elements from evidence data into policy briefs/explicit and oriented notes/knowledge documents
**3 – Identification of a project, image, etc., gathering KT processes to be visible to institutions** *To identify/to use a project/a shared image gathering KT processes for the institution(s)*	**3.1**	**Institutional communication about a KT programme/plan**	Institutional communication about a KT programme/plan in journal publications, during institutional meetings, etc.
	**3.2**	**Use of the KT programme to develop specific partnerships (research, other associations)**	Use of the KT programme/plan to develop specific partnerships, for example, with research teams, other associations, field professionals, etc.
	**3.3**	**Identification of a graphic charter for KT activities**	Identification of a graphic charter for KT activities with the aim that KT activities be easily noticed by publics
	**3.4**	**Evaluation of its KT strategy**	Planning an evaluation of its KT strategy through data collection, interviews, focus groups, observations, etc.
**4 – Communication dedicated to evidence** *Planning communication moments specifically dedicated to evidence*	**4.1**	**Symposium/meeting including specific communication about evidence**	Organising symposium/meetings/presentations that are dedicated to evidence
**5 - Communication on evidence in communications not dedicated to evidence** *Planning/realisation of communications on evidence during communication moments not dedicated to evidence*	**5.1**	**Communication/mention of evidence within meetings not dedicated to evidence**	Communication/mention of evidence within meetings not dedicated to evidence (e.g. meetings, research symposium, presentations, etc. not dedicated to evidence)
	**5.2**	**Inclusion of the interest of evidence (added value) into existing thematic or generalist training courses**	Inclusion of the interest of evidence (added value) into existing thematic or generalist training courses not dedicated to evidence
**6 – Training on evidence use** *Organisation of training sessions on evidence utility, adding value and use*	**6.1**	**Awareness on evidence use (meetings, seminars, etc.)**	Awareness on evidence use, utility and issues on several occasions, for example, during internal/external meetings, seminars, etc.
	**6.2**	**Training on evidence analysis and use**	Training courses dedicated to evidence analysis and use
**7 – Appropriation of evidence** *Exchange and working sessions that foster the identification, sharing and analysis of evidence (they could lead to the production of tools but not necessarily)*	**7.1**	**Analysis and exchange workshops on targeted evidence**	Exchange and working sessions, workshops, etc. that foster the identification, sharing and analysis of evidence (they could lead to the production of tools, nut not necessarily) and therefore lead to evidence appropriation
**8 – Support to evidence use through processes and structures** *Institutional reorganisation to the advantage of KT and EIDM*	**8.1**	**Service/unit/support centre for KT development**	Institutional reorganisation to the advantage of KT and EIDM: development of services/units/support centres into the organisation
	**8.2**	**Service/unit/pole for evaluating promising practices**	Institutional reorganisation to the advantage of KT and EIDM: creation of services/units/support centres for evaluating promising practices
	**8.3**	**Amendment or reinforcement or orientation of the activity of an existing KT plan**	Institutional reorganisation to the advantage of KT and EIDM: Amendment or reinforcement or orientation of the activity of an existing KT plan
	**8.4**	**Internal coordination meetings (how to use evidence?)**	Intra-organisation meetings to talk about evidence usefulness, bibliographic needs, calls for proposal and their evidence requirement, etc.
	**8.5**	**Reminder of the importance (interest, added value) of using evidence/SIPREV during team and/or project meetings**	Reminder of the importance (interest, added value) of using evidence during intra-organisation team and/or project meetings
	**8.6**	**Reminder of the importance (interest, added value) of using evidence/SIPREV in work or financial documents**	Reminder of the importance (interest, added value) of using evidence in work or financial documents (e.g. calls for proposal documents)
**9 – Methodological support to evidence use** *Using specific tools or support field professionals that help to evidence use, analysis and transfer*	**9.1**	**Occasional methodological support including evidence (less than 2 h)**	Occasional methodological support that includes evidence is provided (less than 2 hours)
	**9.2**	**Short methodological support including evidence (2–6 hours)**	Short methodological support that includes evidence is provided (2 to 6 hours)
	**9.3**	**Long methodological support including evidence (more than 6 hours)**	Long methodological support that includes evidence is provided (more than 6 hours)
	**9.4**	**Existence of a proactive referent for KT roll-out: to encourage, mobilise, remind and support KT development**	A proactive referent for KT roll-out is identified into the organisation and systematically encourages, mobilises, reminds and supports KT development in that organisation
	**9.5**	**Methodological support for KT roll-out**	A methodological support for KT roll-out is provided (support more intensive than methodological support which include evidence)
	**9.6**	**Creation and diffusion of methodological tools based on evidence synthesis (grids, referentials) to support evidence synthesis use in an autonomous way**	Methodological tools based on evidence synthesis (grids, referentials) are developed and shared in order to support evidence synthesis use, in an autonomous way
	**9.7**	**Development of a methodological guide to help KT implementation**	Methodological guides to help KT implementation are developed
	**9.8**	**Development of methodological guides to assist in the use of tools developed using evidence (from SIPREV or not)**	Methodological guides to assist in the use of tools developed using evidence (e.g. video capsules evidence-based) are developed
**10 - Co-construction of KT tools** *Approaches to* *develop new shared knowledge*	**10.1**	**Multidisciplinary and multi-professional co-construction of KT tools and processes**	KT tools and processes are developed in a multidisciplinary and multi-professional way
**11 – KT advocacy** *Implementation of advocacy strategies to support EIDM*	**11.1**	**Advocacy to decision-makers**	Advocacy to decision-makers is performed in order to support EIDM
	**11.2**	**Advocacy to partners**	Advocacy to partners is performed in order to support EIDM

*EIDM* evidence-informed decision-making, *KT* knowledge translation, *SIPREV* Stratégies d’Intervention en Prevention (knowledge documents named ‘intervention strategies in prevention’)

## Discussion

Based on a participative process between researchers, decision-makers and stakeholders, we developed a KT taxonomy composed of 35 standardised KT activities grouped into 11 categories. This taxonomy can now be used in local contexts to help develop and assess KT plans in the field of health prevention. Indeed, it may be used to develop KT plans, distinguish some activities in the evaluation purpose, and help to advocate KT policies in the field of health prevention by providing clear examples of activities for decision-makers.

In the taxonomy, there are the three knowledge transfer strategies recognized as the most effective in the scientific literature [12, 17, 25, 31–36]: (1) appropriate access to knowledge, including marketing techniques and reminders (e.g. some activities grouped in the ‘dissemination of evidence’ and ‘adaptation of evidence’ categories), (2) organisation restructuring, including norms and social incentives (e.g. some activities grouped in the ‘communication on evidence in communications not dedicated to evidence’ and ‘support for evidence use through processes and structures’ categories), and (3) stakeholder support (e.g. some activities grouped in the ‘training for evidence use’ and ‘methodological support’ categories). It also fits in with work on different approaches to KT, distinguishing between the knowledge-driven approach (e.g. some activities from the ‘dissemination of evidence’ category), the problem-solving approach (e.g. some activities described in the ‘adaptation of evidence’ and ‘methodological support of evidence use’ categories) and the interactive approach (e.g. some activities from the ‘co-construction of KT tools’ or ‘appropriation of evidence’ categories) [37].

Finally, apart from existing KT frameworks, a wide-ranging review in 2015 identified 51 classification plans for KT interventions to integrate evidence into healthcare practice [20]. They relate to several areas of application (e.g. behavioural change, education, mental health) [38], while only one of them, the classification by Lavis et al. [39], was directly related to KT. This classification has been widely cited in the KT literature [38] and is designed to assess efforts at linking action to research [13, 39]. It distinguishes four clusters of KT activities, namely push efforts (mainly efforts to prepare and communicate evidence briefs to research users and efforts to enhance the capacity of researchers to develop and execute evidence-informed push efforts), efforts to facilitate user-pull (mainly efforts to provide access to research and efforts by researchers to develop research users’ capacity to apply research), user-pull efforts (mainly efforts to facilitate research use and efforts to develop structures and processes to help research users acquire, assess, adapt and apply research), and exchange efforts (including efforts to enhance the capacity of researchers and research users to engage in mutually beneficial partnerships). The framework-like classification provides an overview of KT interventions but lacks consistency when implementing KT plans in local contexts.

In comparison with this classification, the taxonomy we developed is more detailed and provides practical examples of KT activities that can be implemented. The KT activities are adapted to local contexts since they were implemented in the regions before being added to the taxonomy. It should be noted that our taxonomy and the classification by Lavis et al. [39] do not follow the same reasoning, i.e. the standardised KT activities we describe cannot be directly related to any one of the clusters described by Lavis et al. If we take the example of standardised activity 10.1, ‘multidisciplinary development of KT tools and strategies’, this KT activity appears to be simultaneously based on push efforts, efforts to facilitate user-pull, user-pull efforts and exchange efforts, which are complementary. Because this taxonomy is built from the concerns of professionals and describes 35 standardised activities that they know how to implement easily, we argue that it is very relevant to developing KT among professionals. The main added value of the taxonomy is that it provides both content and methods that are easy to implement in local health prevention contexts marked by fewer resources and skills than in other fields (the field of care) or in national policies.

The taxonomy we developed therefore provides practical examples of KT activities, defined in a standardised way, which can help professionals in the field to implement effective KT plans and help evaluators to assess and compare the different activities implemented. The next step in the TC-REG process will be to decide on the best combination of KT activities to support the use of evidence according to local contexts. This work is currently underway, based on a realistic evaluation [2–4]. Realistic approaches aim to identify context–mechanism–outcome (CMO) configurations for a given complex intervention, i.e. how the interactions between contexts and interventions activate specific mechanisms to inform outcomes of interest.

In terms of context, two categories need to be distinguished, namely, those directly related to the intervention (i.e. context-related elements of the intervention) and those not related to the intervention (i.e. pre-existing context elements). In the TC-REG study, KT activities implemented in regions are contextual elements directly related to the intervention. On the other hand, data collected to highlight CMO configurations in realistic evaluations remains challenging [40–42] – how can we compare the activities conducted with precision? The methodological work presented here enables us to compare the KT plans implemented in the four regions in a standardised manner. It provides a practical example of how contextual data related to the interventions can be collected and standardised according to different settings. Our taxonomising approach can help realistic evaluators to better determine the components required when assessing CMO configurations.

### Strengths and limitations

The main strength of this work is the rigorous methodology used to develop the taxonomy. We prepared a seminar based on effective existing KT strategies, supported by the well-known work of Langer et al. [25], which combined systematic and social science reviews. Discussion of the findings between decision-makers and field professionals involved in the TC-REG project helped them to develop and implement KT plans that were adapted to local contexts and based on effective KT strategies. Field data was gathered on the implementation of KT plans, leading to the development of a consensually agreed KT taxonomy between researchers, decision-makers and field professionals working in the health promotion and health prevention sectors in France. Moreover, we adopted a participative and empirical methodology involving researchers and experts in public health and health prevention settings (decision-makers, field professionals and researchers) to make it more robust. The taxonomy we developed was based on data collected after the implementation of evidence-based KT plans, developed through a multidisciplinary approach. Thus, the activities described in the taxonomy are feasible and consider some forms of activity not found in the literature. In addition, the present work provides a useful tool to help professionals from the public health and health prevention sector to develop and evaluate KT plans in local contexts through a precise description of the activities implemented, in line, for example, with behavioural change techniques in another field [43].

However, the work also has some limitations. The first is that the taxonomy was developed in a French and local context. Other strategies may potentially be used at national level, for example, or in other countries where the organisation of professionals in the field of health prevention may be different. We believe that some activities we did not take into consideration may well be implemented elsewhere and could be added to the taxonomy. We are fully aware that other elements could be added, and therefore recommend further research to test its usability in other contexts as well as the most effective combinations of these activities. The second limitation is that, while we could have conducted a systematic review to prepare the seminar, we preferred to use a single, although valuable, summary of such a review for reasons of time and resources. Perhaps other literature reviews could provide further examples of strategies other than those in Langer et al.’s [25] review and these could be included in a future taxonomy.

## Conclusion

The work described in this article offers a first step towards developing more evidence-based decision-making and practices in the public health sector. It offers a KT taxonomy built on a participative approach between researchers, field professionals and decision-makers, based on both data from the literature and field practice. On the other hand, it needs further content input if it is to be used internationally. The taxonomy appears to be a promising tool for developing and evaluating KT plans in local contexts in the field of health prevention. The next step is to test its usability in other contexts. In KT research, we argue that this kind of taxonomy could help to provide operational guidance for local health prevention authorities to implement KT strategies and evaluate and compare KT strategies. It also illustrates the determination of CMO configurations that have to be assessed through a realistic assessment. Indeed, this approach develops a consideration of the context of implementation as a key factor. Establishing a taxonomy of these elements allows us to compare specific factors without confusion (different activities using the same term or different terms for the same activity). The next step in TC-REG is to explore the best combinations of these activities, a process that is currently underway.

## Data Availability

All the data generated or analysed during this study are included in the paper.

## Abbreviations

ARS: Agence Régionale de Santé
CMO: Context–Mechanism–Outcomes
EIDM: evidence-informed decision-making
IREPS: Instance Régionale d’Education et de Promotion de la Santé; Regional Authority of education and health promotion)
KT: knowledge translation
TC-REG: Transfert de connaissances en region, Knowledge translation in regions
